# Use of ICD-9-CM coding for identifying antibiotic prescriptions during hospitalization: a Delphi consensus model

**DOI:** 10.1016/j.infpip.2024.100416

**Published:** 2024-10-28

**Authors:** Agnese Comelli, Camilla Genovese, Giulia Renisi, Luigia Scudeller, Martina Zanforlini, Giulia Macaluso, Arianna Mazzone, Antonio Muscatello, Giorgio Bozzi, Alessia Zoncada, Angelo Pan, Marianna Rossi, Paolo Bonfanti, Stefania Chiappetta, Salvatore Casari, Marco Ripa, Antonella Castagna, Liana Signorini, Francesco Castelli, Margherita Chiamenti, Giulia Carla Marchetti, Barbara Castiglioni, Fabio Franzetti, Elena Graziano, Paolo Grossi, Paola Morelli, Michele Bartoletti, Chiara Molteni, Stefania Piconi, Marco Merli, Massimo Puoti, Davide Ricaboni, Luigi Pusterla, Chiara Cerri, Angelo Regazzetti, Laura Soavi, Marco Rizzi, Marco Franzetti, Stefano Rusconi, Erika Asperges, Raffaele Bruno, Monica Schiavini, Andrea Gori, Simone Schiatti, Alessandra Bandera

**Affiliations:** a Infectious Diseases Unit, Foundation IRCCS Ca’ Granda Ospedale Maggiore Policlinico, Milan, Italy; b Department of Biomedical and Clinical Sciences, University of Milan, Milan, Italy; c Scientific Direction, Fondazione IRCCS Policlinico San Matteo, Pavia, Italy; d Regional Company for Innovation and Purchasing Aria S.p.A., Milan, Italy; e Infectious Diseases, ASST di Cremona, Cremona, Italy; f Infectious Diseases Unit, Foundation IRCCS San Gerardo dei Tintori, Monza, Italy; g Department of Medicine and Surgery, University of Milano-Bicocca, Milan, Italy; h Infectious Diseases Unit, Carlo Poma Hospital, ASST Mantova, Mantua, Italy; i Infectious Diseases Unit, IRCCS San Raffaele Scientific Institute, Milan, Italy; j Department of Infectious Diseases, Vita-Salute San Raffaele University, Milan, Italy; k University Department of Infectious and Tropical Diseases, ASST Spedali Civili, Brescia, Italy; l Department of Health Sciences, Clinic of Infectious Diseases, ASST Santi Paolo E Carlo, Milan, Italy; m Infectious Diseases Unit, ASST Valle Olona, Busto Arsizio Hospital, Busto Arsizio, Italy; n University Department of Infectious and Tropical Diseases Unit, ASST Sette Laghi, Varese, Italy; o Infectious Disease Unit, IRCCS Humanitas Research Hospital, Rozzano, Italy; p Infectious Diseases Unit, Alessandro Manzoni Hospital, ASST Lecco, Lecco, Italy; q Infectious Diseases, Hospital Niguarda, Milan, Italy; r Division of Infectious Diseases, ASST Lariana, Como, Italy; s UOC Di Malattie Infettive E Tropicali - Ospedale Delmati di Sant’Angelo Lodigiano, ASST Di Lodi, Lodi, Italy; t Infectious Diseases Unit, ASST Papa Giovanni XXIII, Bergamo, Italy; u Infectious Diseases Unit, Ospedale Civile di Legnano, ASST Ovest Milanese, Legnano, Italy; v Infectious Diseases, Fondazione IRCCS Policlinico San Matteo, Pavia, Italy; w Department of Infectious Diseases, Unit II, L. Sacco Hospital, ASST Fatebenefratelli Sacco, Milan, Italy

**Keywords:** Antimicrobial prescription, ICD9-CM, AMS

## Abstract

A Delphi consensus-seeking procedure was conducted to validate a list of ICD-9-CM codes that could help identify hospital admissions in which antimicrobials are more likely to be prescribed. The panel agreed to include 2967 codes out of 16229 (18.28%). Such codes could support AMS strategies by large-scale monitoring of drug consumption.

## Background

Globally, in 2019, antimicrobial resistance (AMR) contributed to an estimated 4.95 million deaths and almost 48 million disability-adjusted life years (DALYs) [1]. Besides, AMR carries significant economic costs, mainly related to its associated morbidity and higher resource utilisation. In 2017, the World Bank estimated that by 2050, AMR will contribute to a 3.8% loss of the world's annual gross domestic product (GDP) [2]. One of the major risk factors recognised for the development and diffusion of antimicrobial-resistant bacterial strains (MDROs) is inappropriate antibiotic prescribing and consumption [3]. Antimicrobial consumption is, in fact, key data for planning antimicrobial stewardship (AMS) programs, targeting education and monitoring the effect of AMS programs carrying out big data analysis, including prevalence and economic evaluations.

Antimicrobial consumption is usually monitored and transmitted to central authorities through centralised informative systems that track hospital prescriptions. Yet in several countries, like Italy, drug prescription systems are rarely computerised and, if they are, data on antimicrobial consumption are seldom transmitted to dedicated institutions.

In such settings, using hospital health records (HDR) that are routinely transmitted to central authorities may be the only way to monitor antimicrobial consumption at the regional/national level.

Indeed, the International Classification of Diseases (ICD), Ninth Revision, Clinical Modification (ICD-9-CM), which was developed and adopted worldwide to categorise and assign codes to diagnoses and procedures associated with hospitalisation, has a crucial importance [4]. Other than for clinical purposes, the ICD-9-CM is widely used in public health programs to identify health conditions of interest through aggregated data. The current ICD-10-CM, instead, was developed in the early 2010s to adapt to changes in the healthcare field [5,6]. Even though the ICD-10 is used for mortality statistics in more than 100 countries worldwide, it has been adopted for morbidity purposes in few countries [7,8]. In Italy, as of the beginning of 2024, the ICD-9-CM is currently in use [9].

Therefore, the ICD codes could help to identify hospital admissions in which antimicrobials are more likely to be prescribed. However, basing this analysis solely on infection-related ICD codes (e.g., the first chapter of ICD-9-CM) could underestimate the real number of hospitalisations associated with antimicrobial prescriptions.

Even though few studies have analysed the appropriateness of community-based antibiotic prescribing by associating the ICD-9-CM code used for diagnosis to drug dispensing records [10,11], classification of ICD codes was proposed by the authors, but did not go through a process of validation. One possible way to validate such classifications could be to carry out Delphi consensus-procedures, which are commonly used in scientific literature to obtain validation among experts about a specific issue [12].

Considering this, we aimed at developing and further validating, through a Delphi consensus process, a model to identify ICD-9-CM codes that may be associated with hospital antimicrobial administration.

## Methods

### Delphi process

A Delphi consensus-seeking procedure was conducted to validate a list of ICD-9-CM codes that could identify clinical conditions that require oral/intravenous (IV) antimicrobial treatment during hospital admission.

As a first step, all of the 16,229 ICD-9-CM codes (adult and paediatric), including the COVID-19 codes added in 2020, were independently reviewed by two infectious diseases (ID) specialists from IRCCS Fondazione Ca’ Granda Ospedale Maggiore Policlinico of Milan. Each code was either classified as “for inclusion” or “for exclusion” based on the following definition: “clinical condition for which it can be assumed that an antibiotic or antifungal systemic therapy (intravenous or oral) was administered during hospital stay in at least 60% of cases”. Based on this definition, antimicrobial prophylaxes and topical antimicrobial therapies were excluded. A third reviewer resolved discrepant classifications.

Subsequently, a panel of ID specialists from the Lombardy ID Network was recruited in a Delphi procedure. This network involves 18 ID units from different public and private hospitals in Lombardy, Italy [13]. An email invitation to participate was sent to one senior representative from each of the 18 ID units. Upon approval, participants were included, and an email with precise instructions was delivered.

At least two rounds were expected; for each round, feedback from the participants was sent to the research team exclusively.

In the first round, participants were asked to use a 9-point Likert scale (1= totally disagree, 9= totally agree) to score their agreement on each of the 16,221 proposed classifications of ICD9-CM codes. For scores <7 (i.e. moderate or poor agreement), participants could add comments or references to justify their disagreement. Missing responses were actively sought from the participants until completion.

Further rounds were proposed for the codes that did not reach an agreement, defined as follows: i) codes with a median score between 4 and 6 or ii) codes with a median score between 7 and 9 with an interquartile range (IQR) greater than the inter-percentile range adjusted for symmetry (IPRAS) [14]. Instead, codes with a median score <4 or ≥7 (with an IQR smaller than the IPRAS) were not subjected for further rounds as agreement was reached.

Rounds continued until an agreement was obtained for all the ICD-9-CM codes.

### Statistical analysis

Descriptive statistics were employed: numbers and percentages were reported.

## Results

All 18 ID units participated in the Delphi consensus-seeking procedure.

Characteristics of the hospital network have already been presented in a previous publication [13].

A total of two rounds were required to reach an agreement for all the proposed codes.

In the first round, full agreement was reached for 16,158 codes: 2,925 as “for inclusion” and 13,233 as “for exclusion”.

Results for some common gastrointestinal infection codes are shown in Table I.

**Table I:**
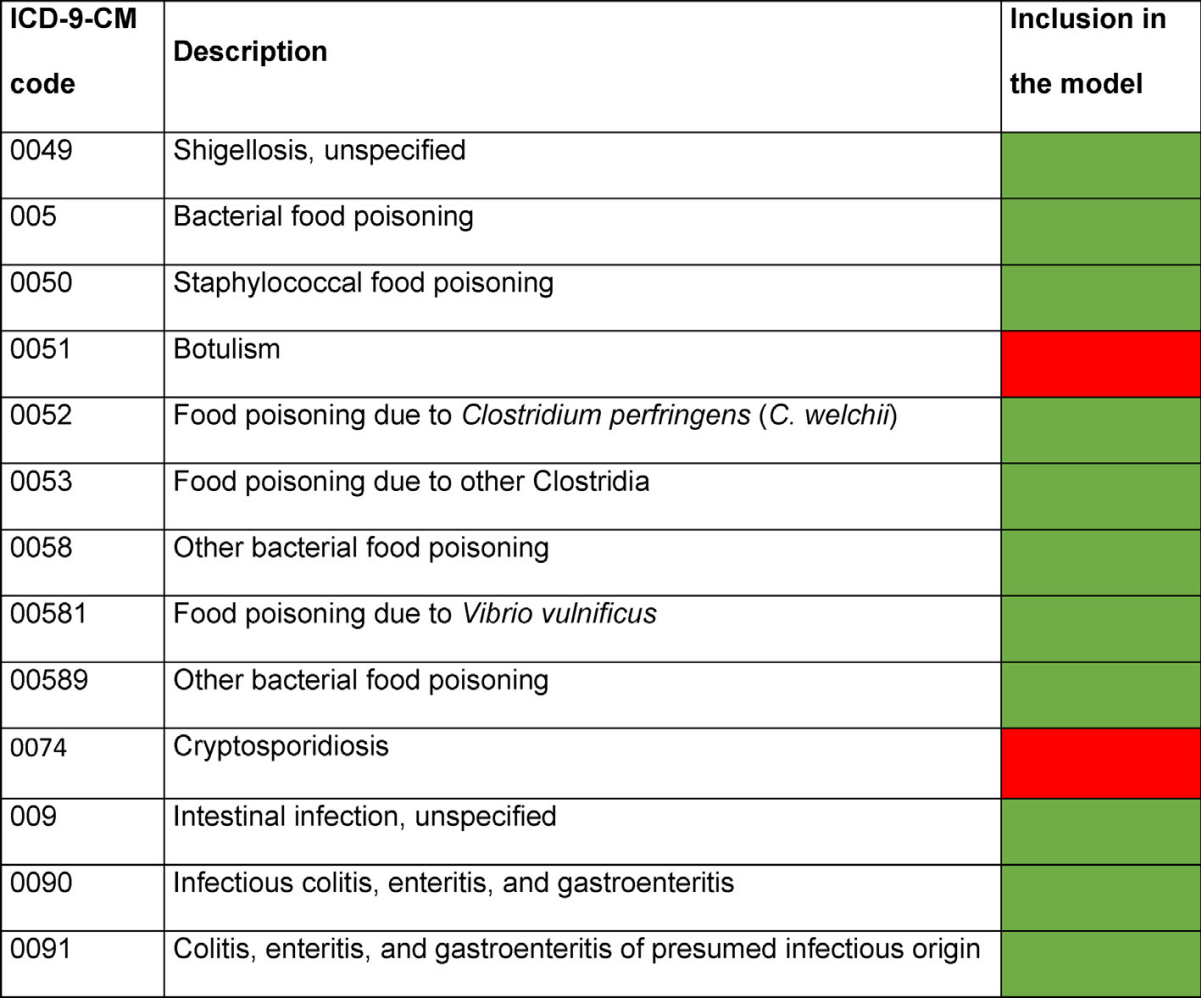
Delphi results for common gastrointestinal infections related ICD-9-CM codes

**Figure 1:**
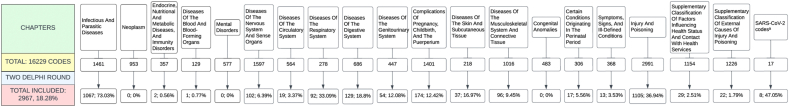
Flow chart of ICD9-CM coding inclusion process. ^a^ SARS-CoV-2 codes were added in the year 2020.

Of the 69 codes where agreement was not found, 39 (56.5%) belonged to Infectious and Parasitic Diseases (IPD) Chapter, 10 (14.5%) to Diseases of The Skin And Subcutaneous Tissue Chapter, 9 (13%) to Diseases of The Respiratory System Chapter, 3 (4.4%) to Diseases of The Nervous System And Sense Organs Chapter, 3 (4.4%) to Supplementary Classification of Factors Influencing Health Status and Contact With Health Services Chapter and 1 for each of remaining chapters (Figure 1).

The panel agreed in the first round to include 8 out of 17 (47.05%) SARS-CoV2-related codes (Table S1).

In the second round, full consensus on the 69 codes reproposed for voting was reached by all participants.

In conclusion, 2,967 ICD-9-CM codes (18.3%) were classified as “for inclusion” (Table S2).

The most represented codes belonged to the Injury and Poisoning Chapter (37.2%,1105/2967), IPD Chapter (36%,1067/2967), and Complication of Pregnancy, Childbearing and the Puerperium Chapter (5.9%,174/2967).

## Discussion

AMS programs, through coherent sets of actions promoting responsible use of antimicrobials [15], play a crucial role in contrasting MDROs diffusion and are associated with a significant reduction in antimicrobial consumption and hospital length of stay without having a negative impact on mortality [16]. Eventually these interventions aim also to reduce healthcare costs [17,18].

Indeed, when planning antimicrobial stewardship programs, antibiotic consumption data are critical to inform strategies. Those data help carry out big data analysis, such as prevalence and economic evaluations that may guide/target educational and/or restrictive activities. Antimicrobial consumption, however, is often monitored through centralised informative systems that track hospital prescriptions. Unfortunately, drug prescription systems are rarely computerised in several countries, making monitoring even more complicated to perform. In these cases, the use of HDR may represent the only solution to monitor healthcare events at the regional/national level.

Therefore, we built a model that included ICD-9-CM codes that could detect hospital antimicrobial administration by identifying hospital admissions in which antimicrobials are more likely to be prescribed.

Using a Delphi process, our model has benefited from the expertise of 18 ID specialists working in large public and private hospitals, with 94.5% working in hospitals with more than 500 beds [13] and covering a catchment population of around 10 million people. To our knowledge, this is the first time ICD codes have been used to build a model to estimate drug consumption. By contrast, this coding has been used to estimate the incidence of sepsis and hospital-acquired infection (HAI), even if with controversial results [[19], [20], [21]], and the appropriateness of antibiotic prescribing [10,11]. In fact, in a recent paper, Leslie *et al.* linked ICD-9-CM codes used for billing purposes to drug dispensing records using a previously published schema where to each of the ICD-9-CM codes a degree of indication for antibiotic treatment in the community setting was assigned [10,11]. However, such a study was designed for community settings and could not be carried out in settings in which drug dispensing records are not centralised.

It could be argued that this estimation could be achieved by simply considering ICD-9-CM codes belonging to the IPD Chapter. However, antibiotics are usually prescribed empirically for conditions outside the ID chapter, and some infectious diseases are included in other chapters, such as meningitis in the Diseases of The Nervous System and Sense Organs Chapter or peritonitis in the Diseases of The Digestive System Chapter. In fact, in our model, most of the included codes do not belong to the IPD Chapter, but to the Injury and Poisoning one, which includes many traumatic conditions that usually require an antibiotic prescription, such as burns, amputation, and necrosis processes. On the other hand, not all conditions in the IPD chapter require antibiotic treatment.

Since HDR reporting is standardised, our tool might be used for research and public health purposes. Firstly, it can help identify a set of HDR for which some indicators, such as microbiological isolates and resistance patterns, can be studied before and after the introduction of a new ASP. In the same way, the model can assist in the economic evaluation of an ASP in a hospital network. Lastly, it can help health authorities identify hospitals where greater consumption of antibiotics may take place because of the type of hospital admissions, e.g. hospitals which host a great number of burns and trauma.

The present model, however, has several limitations.

The HDR coding process, a crucial part of the model, is highly subjective and is currently done manually by medical doctors responsible for patient discharge, with significant variability in the pattern of choices. Secondly, the model is based on an outdated coding system (ICD9-CM) that will be replaced by a more updated one (e.g. ICD-10 and ICD-11) in the near future. However, tools are available to help with mapping between different coding systems and could be used to transition from ICD9-CM to new coding systems [22]. Also, the model reflects the prescription attitude of a group of ID specialists in a single region and may be biased towards a common pattern of clinical case presentation and shared protocols and guidelines. Therefore, to validate its use and improve its applicability at national level, the model's sensitivity and specificity compared to actual drug consumption in different hospital settings must be analysed.

To obtain more time-consistent HDR data without the influence of COVID-19 hospital admissions, the model's sensitivity and specificity analysis will be the aim of a future dedicated study with a suitable number of HDR in different periods (pre-COVID years, 2020–2022, 2023 onward).

## Conclusion

In the future, electronic patient charts and drug prescriptions will allow healthcare authorities to gather data on drug consumption. However, this approach may only be feasible for some high-income countries, while this model may remain a valuable tool for most middle-income countries and some high-income countries. Therefore, a validated model using HDR to monitor drug consumption, both at the local and national levels, will be useful in supporting large-scale analyses for both public health measures and antimicrobial stewardship strategies.

## Credit author statement

Agnese Comelli, Camilla Genovese conceptualization, Data curation, Methodology, Investigation, Writing original draft, Giulia Renisi Investigation, Luigia Scudeller Formal analysis, Methodology and Writing - review and editing, Martina Zanforlini, Giulia Macaluso, Arianna Data curation, Alessandra Bandera Supervision, Validation, Writing - review and editing. Alessia Zoncada, Angelo Pan, Marianna Rossi, Paolo Bonfanti, Stefania Chiappetta, Salvatore Casari, Marco Ripa, Antonella Castagna, Liana Signorini, Francesco Castelli, Margherita Chiamenti, Giulia Carla Marchetti, Barbara Castiglioni, Fabio Franzetti, Elena Graziano, Paolo Grossi, Paola Morelli, Michele Bartoletti, Chiara Molteni, Stefania Piconi, Marco Merli, Massimo Puoti, Davide Ricaboni, Luigi Pusterla, Chiara Cerri, Angelo Regazzetti, Laura Soavi, Marco Rizzi, Marco Franzetti, Stefano Rusconi, Erika Asperges, Bruno Raffaele, Monica Schiavini, Simone Schiatti and Andrea Gori Investigation, Validation. All authors approved the final version of the manuscript.

## Ethics

Non required.

## Funding

Supported by Italian Ministry of Health/Lombardy Region project RF NET 2018 12366982-4.

## Conflict of interest

None.
