# Supplementary material for: Use of ICD-9-CM coding for identifying antibiotic prescriptions during hospitalization: a Delphi consensus model
Source: Infect Prev Pract. 2024 Oct 28;6(4):100416. doi: 10.1016/j.infpip.2024.100416 (PMC11651032; doi:10.1016/j.infpip.2024.100416)
Supplement: Multimedia component 1 [file mmc1.docx]

Table S1. Delphi results for SARS-CoV2 related ICD-9-CM codes

| ICD-9-CM code | Description | Inclusion in the model |
| --- | --- | --- |
| 04311 | COVID-19 established, virus identified |  |
| 04312 | COVID-19 established, virus not identified |  |
| 04321 | Paucisymptomatic COVID-19, virus identified |  |
| 04322 | Paucisymptomatic COVID-19, virus not identified |  |
| 04331 | COVID-19 asymptomatic, virus identified |  |
| 04332 | COVID-19 asymptomatic, virus not identified |  |
| 48041 | Pneumonia in COVID-19, virus identified |  |
| 48042 | Pneumonia in COVID-19, virus not identified |  |
| 51891 | Respiratory distress syndrome (ARDS) in COVID-19, virus identified |  |
| 51892 | Respiratory distress syndrome (ARDS) in COVID-19, virus not identified |  |
| 51971 | Other respiratory tract infection in COVID-19, virus identified |  |
| 51972 | Other respiratory tract infection in COVID-19, virus not identified |  |
| V0185 | Exposure to SARS-CoV-2 |  |
| V0700 | Isolation needed to SARS-CoV-2 related risk |  |
| V0708 | Other need of isolation |  |
| V1204 | Personal history of SARS-CoV-2 infection (COVID-19) |  |
| V7184 | Observation and evaluation for suspected exposure to SARS-CoV-2 |  |
